# Conversion between two conformational states of KaiC is induced by ATP hydrolysis as a trigger for cyanobacterial circadian oscillation

**DOI:** 10.1038/srep32443

**Published:** 2016-09-01

**Authors:** Katsuaki Oyama, Chihiro Azai, Kaori Nakamura, Syun Tanaka, Kazuki Terauchi

**Affiliations:** 1 Graduate School of Life Sciences, Ritsumeikan University, Kusatsu, Shiga 525-8577, Japan; 2 College of Life Sciences, Ritsumeikan University, Kusatsu, Shiga 525-8577, Japan

## Abstract

The cyanobacterial circadian oscillator can be reconstituted *in vitro* by mixing three clock proteins, KaiA, KaiB and KaiC, with ATP. KaiC is the only protein with circadian rhythmic activities. In the present study, we tracked the complex formation of the three Kai proteins over time using blue native (BN) polyacrylamide gel electrophoresis (PAGE), in which proteins are charged with the anionic dye Coomassie brilliant blue (CBB). KaiC was separated as three bands: the KaiABC complex, KaiC hexamer and KaiC monomer. However, no KaiC monomer was observed using gel filtration chromatography and CBB-free native PAGE. These data indicate two conformational states of KaiC hexamer and show that the ground-state KaiC (gs-KaiC) is stable and competent-state KaiC (cs-KaiC) is labile and degraded into monomers by the binding of CBB. Repeated conversions from gs-KaiC to cs-KaiC were observed over 24 h using an *in vitro* reconstitution system. Phosphorylation of KaiC promoted the conversion from gs-KaiC to cs-KaiC. KaiA sustained the gs-KaiC state, and KaiB bound only cs-KaiC. An E77Q/E78Q-KaiC variant that lacked N-terminal ATPase activity remained in the gs-KaiC state. Taken together, ATP hydrolysis induces the formation of cs-KaiC and promotes the binding of KaiB, which is a trigger for circadian oscillations.

The circadian clock is an endogenous timing mechanism in living organisms that coordinates various biological activities with daily environmental changes^1^. Cyanobacteria are the simplest organisms that generate circadian rhythms^2^, and the three clock genes *kaiA*, *kaiB* and *kaiC* are essential for circadian clock activities in the cyanobacterium *Synechococcus elongatus* PCC 7942^3^. The circadian clock of *S. elongatus* PCC 7942 is currently the only circadian system that can be reconstituted *in vitro*, and the circadian oscillation of KaiC phosphorylation can be reconstituted by mixing KaiA, KaiB and KaiC with ATP *in vitro*^4^. This oscillator has been analysed as a model of a self-sustaining biological clock and provides a unique model for sub-molecular studies of biological clocks. Moreover, this biochemical oscillator satisfies the three criteria of circadian clocks^4^,^5^, and rhythmic behaviours of the oscillator have been confirmed in many functional and structural analyses. Physiological characteristics of the circadian system reflect molecular features in the Kai protein oscillator, including temperature-compensated KaiC ATPase, autophosphorylation and autodephosphorylation activities^4^,^6^. The main oscillator protein, KaiC, forms a homo-hexamer^7^,^8^ and the intra-hexameric interactions are essential for generation of circadian rhythm; the three activities of KaiC are regulated each other by specific interactions between the protomers in the hexamer^9^.

KaiC comprises two homologous N-terminal CI and C-terminal CII domains, and both domains possess P-loop ATPase motifs^10^. X-ray crystallographic structure analyses revealed that KaiC assembles as a ring-like *C*_6_-symentrical hexamer with two ATP molecules at each protomer interface^8^. KaiC has autophosphorylation and autodephosphorylation activities in addition to ATPase activity^11^,^12^,^13^, and the two residues Ser431 and Thr432 are proximal to the protomer interface in the CII-domain and act as phosphorylation sites^14^. Phosphorylation states of KaiC and associations with KaiA and KaiB define the phase of circadian timing. In particular, KaiA stimulates KaiC phosphorylation by repeated association with KaiC, and Thr432 and Ser431 residues are sequentially phosphorylated in this order^15^,^16^. KaiB interacts with KaiC in a Ser431 phosphorylation-dependent manner, resulting in a switch back to autodephosphorylation^15^,^16^, which is mediated by ATP/ADP phosphotranferase activities of KaiC^17^,^18^,^19^. Binding of KaiB to KaiC is thus a crucial step for generating circadian rhythms^15^,^16^,^20^.

The ATPase activity of KaiC has been proposed as the basic reaction for circadian periodicity^6^,^21^. To keep the circadian oscillation, which is a very slow reaction in chemical senses, a single KaiC protomer hydrolyzes only ~15 ATP molecules per day^6^. While a small part of ATP serve as the phosphate donor of the phosphorylation sites, hydrolysis of most ATP molecules may cause a subtle conformational change in the KaiC hexameric structure, which could provide a structural basis for pace making of circadian oscillation of KaiC^6^. Moreover, similar structural changes of KaiC during the ATPase reaction cycle were proposed to lead to the formation of a stable KaiB–KaiC complex^20^,^22^. Nuclear magnetic resonance (NMR) spectroscopy^23^ and small-angle X-ray scattering (SAXS)^24^ were used to observe the dynamic process of KaiC and provided insights into the conformational changes of KaiC. However, these techniques require high non-physiological concentrations of proteins and expensive and specialised equipment. In the present study, we applied blue native (BN) polyacrylamide gel electrophoresis (PAGE) to track the dynamics of Kai protein assembly and to investigate higher-order structural changes of KaiC during circadian oscillations. BN-PAGE analyses are performed using the mild anionic dye Coomassie brilliant blue (CBB) to confer negative charges to the protein instead of the destructive detergent SDS^25^,^26^. CBBs bind non-specifically to each protein at a nearly constant charge/mass ratio and enable electrophoretic separation of native proteins according to their apparent molecular weights. Two conformational states of KaiC were discriminated using BN-PAGE, including the stable CBB-resistant KaiC hexamer in the ground state (gs-KaiC) and the labile KaiC hexamer in the competent state (cs-KaiC), which easily dissociates to monomers by CBB binding. The results suggest that conversion from gs-KaiC to cs-KaiC is induced by ATP hydrolysis and triggers periodic assembly of Kai proteins during circadian cycles.

## Results

### Dynamics of Kai proteins in BN-PAGE

**Figure 1:**
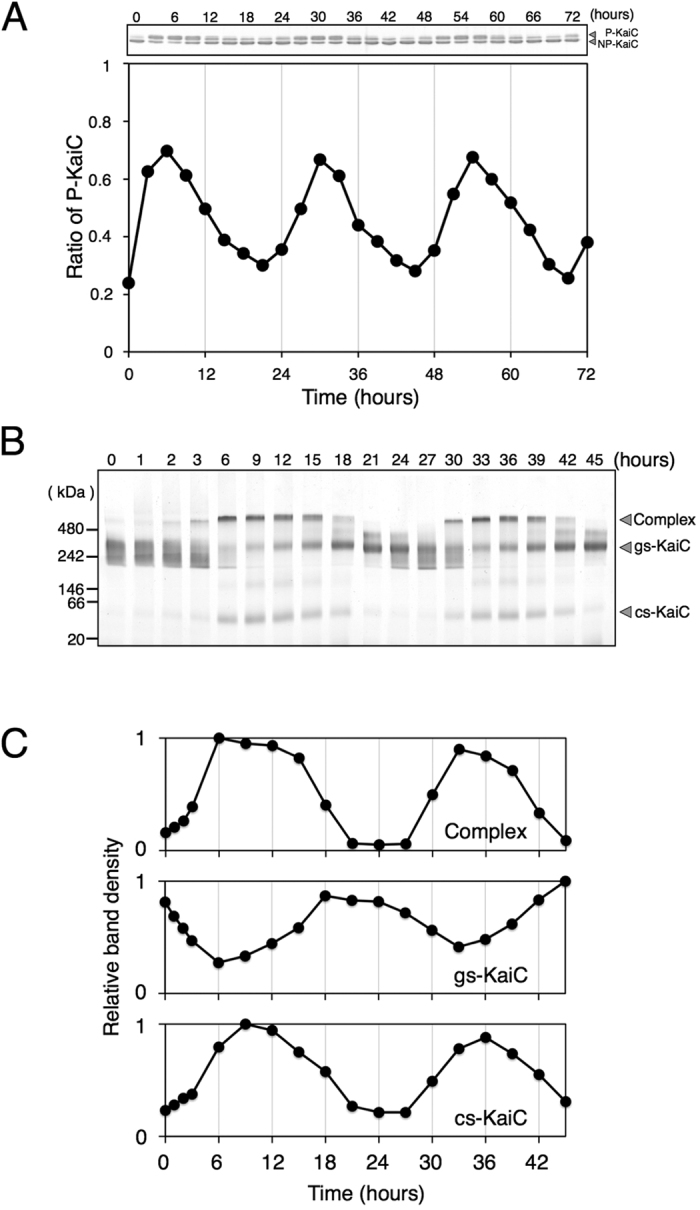
BN-PAGE analysis of Kai proteins in the *in-vitro* reconstitution system. (**A**) KaiC was incubated with KaiA and KaiB in the presence of ATP for 72 h. Reaction mixtures were then subjected to SDS-PAGE every 3 h (upper panel). In the SDS-PAGE gel, upper and lower bands correspond with phosphorylated (P-KaiC) and non-phosphorylated KaiC (NP-KaiC), respectively. Ratios of P-KaiC to total KaiC were plotted against incubation times (lower panel). The data are representative of more than three experiments. (**B**) KaiC was incubated with KaiA and KaiB in the presence of ATP for 45 h, and aliquots of mixtures were subjected to BN-PAGE analyses every 3 h except for the first 3 h. (**C**) Densitometric analyses of band in (**B**); Observed densities were normalised to maximal densities of each band and were plotted against incubation times. The data are representative of three experiments.

We purified the proteins KaiA, KaiB and KaiC as Strep-tagged proteins and initially confirmed circadian oscillations of KaiC. The reaction mixture contained the three Kai proteins and ATP and was maintained at 4 °C until transfer to 30 °C to start the oscillation reaction. In agreement with a previous study^4^, KaiC phosphorylation levels oscillated over 24 h (Fig. 1A), confirming that the purified strep-tagged proteins were functional.

BN-PAGE has been widely used to resolve protein complexes^25^,^26^. We applied this technique to the analyses of assembly and disassembly of Kai protein complexes in an *in-vitro* reconstitution system (Fig. 1B). Prior to this analysis we analysed individual Kai protein and confirmed very faint bands of KaiA and KaiB in BN-PAGE (Fig. S1). In contrast, KaiC gave two major bands and a faint minor band in BN-PAGE, allowing investigation of the dynamics of KaiC-containing complexes (Fig. S1).

Only one broad band with an apparent molecular mass of 300–400 kDa was detected at the beginning of reconstitution (gs-KaiC in Fig. 1B). Moreover, the apparent molecular weight of this protein was consistent with the predicted weight of homohexameric KaiC (357 kDa). During the first 6 h, the intensity of this diffuse hexamer band decreased and a sharp band with a higher molecular weight of approximately 500 kDa appeared (Complex in Fig. 1B). Subsequent immunoblotting experiments were used to identify the constituents of this band and indicated formation of a KaiA–KaiB–KaiC complex (Fig. S2), which gradually decreased after 6 h with increases in the sharp KaiC hexamer band, and became undetectable at 21 h. In addition, a band with an apparent molecular weight of approximately 50 kDa was observed at 6–18 h (cs-KaiC in Fig. 1B). This molecular weight is consistent with the predicted value for monomeric KaiC (58 kDa), suggesting that some KaiC hexamers dissociated into monomers during BN-PAGE. Similar dynamics were observed during the first and subsequent 24-h periods (Fig. 1B), and densitometric quantification of the three KaiC-containing bands clearly showed circadian oscillation dynamics (Fig. 1C). Moreover, bands corresponding to the KaiA–KaiB–KaiC complex and monomeric KaiC (cs-KaiC) were generated during the phase in which dephosphorylation of KaiC starts (Fig. 1A,C).

### CBB-binding discriminates the two conformational states of the KaiC hexamer

**Figure 2:**
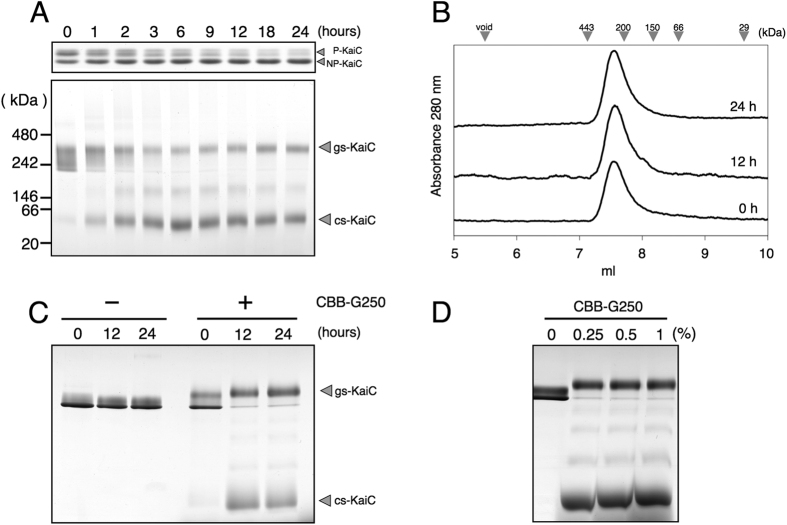
The effect of CBB G250 on KaiC hexamers. (**A**) KaiC was incubated in the presence of ATP at 30 °C for 24 h, and aliquots were subjected to SDS-PAGE (upper panel) and BN-PAGE (lower panel) analyses. (**B**) Elution profiles of KaiC at 0, 12 and 24 h were generated from gel filtration chromatography experiments. Arrowheads at the top of the panel indicate elution volumes of molecular mass standards. (**C**) KaiC was incubated in the presence of ATP at 30 °C, and aliquots were subjected to CBB-free native-PAGE at 0, 12 and 24 h with or without 0.25% CBB. (**D**) KaiC was incubated in the presence of ATP at 30 °C for 12 h, and was subjected to CBB-free native-PAGE following addition of CBB (0%, 0.25%, 0.5% and 1%).

To investigate KaiC dynamics, KaiC was incubated alone at 30 °C and was then subjected to SDS-PAGE and BN-PAGE. In the absence of KaiA–KaiB, phosphoryation levels of KaiC were decreased as previously demonstrated^4^ (Fig. 2A, upper panel). Moreover, BN-PAGE analyses showed KaiC as a broad 300–400 kDa band at the beginning of the incubation (Fig. 2A, lower panel). Another faint band of approximately 50 kDa was also detected, and according to apparent molecular weights these bands were assigned to hexameric and monomeric KaiCs, respectively. During incubation at 30 °C, the lower side of the broad hexameric band disappeared during the first 6 h, and the monomeric band gradually increased concomitantly (Fig. 2A). Hence, some KaiC hexamers dissociated into monomers in BN-PAGE analyses, even under KaiC-only conditions. Moreover, another faint band appeared between the original two bands and became constant until 6 h at 30 °C, implying that the dissociation of KaiC hexamers proceeds via an intermediate Xmeric state.

To confirm that dissociation of KaiC hexamers occurs under physiological conditions, KaiC was subjected to gel filtration chromatography at 30 °C using HPLC with a column packed with silica-based resin (Fig. 2B). In contrast with BN-PAGE analyses, KaiC was eluted as a single peak with an apparent molecular weight of 254 kDa at all time points. Although the estimated molecular weight was significantly smaller than the predicted molecular weight for hexameric KaiC (357 kDa), the same preparation of KaiC eluted from a Sephacryl S-300 column as a single peak with a higher molecular weight of 319 kDa at 4 °C. This molecular weight estimate was in good agreement with those reported previously^7^,^27^. Taken with previously reported gel filtration chromatography experiments, the present gel filtration chromatography peak at 30 °C indicated a hexameric KaiC. Moreover, monodispersed and stable single peaks indicated that KaiC hexamers did not dissociate into monomers under these conditions (Fig. 2B). However, gel filtration chromatography and BN-PAGE analyses gave inconsistent indications of the oligomeric states of KaiC.

During BN-PAGE analyses, proteins are charged and stained with the anionic dye CBB before electrophoretic separation. Hence, binding of CBB may have caused the dissociation of KaiC hexamers into monomers. To investigate this possibility, KaiC was incubated for 24 h at 30 °C and was then electrophoresed with or without 0.25% CBB in a native poly-acrylamide gel. In the absence of CBB, KaiC migrated as a tight band at the same positions at all time points, whereas the band at the 0-h time point was split into the other two major bands following addition of CBB at 12 and 24 h (Fig. 2C). These split bands closely resembled the two major bands observed in BN-PAGE analyses of KaiC (Fig. 2A), suggesting that some KaiC hexamers dissociate because of the binding of CBB. In further experiments with higher concentrations of CBB (0.5% and 1%; Fig. 2D), 0.25% CBB was sufficient for KaiC hexamer dissociation and higher concentrations of CBB gave similar results (Fig. 2D). Moreover, NaCl and other detergents such as dodecyl maltoside, digitonin, cholate and CHAPS had no effects on the dissociation of KaiC hexamers (Fig. S3).

Taken together, the present data indicate that KaiC forms two types of hexamers with different conformational states, including a stable complex that is resistant to CBB-binding and a labile complex that readily dissociates into monomers after CBB-binding. Hereafter, we refer to these KaiCs as ground state KaiC (gs-KaiC) and competent state KaiC (cs-KaiC), respectively. Hence, the present BN-PAGE analyses discriminated between the two conformational states of KaiC as separate bands on gels, which could not be accomplished using gel filtration or CBB-free native-PAGE.

### State conversion of KaiC correlates with phosphorylation

**Figure 3:**
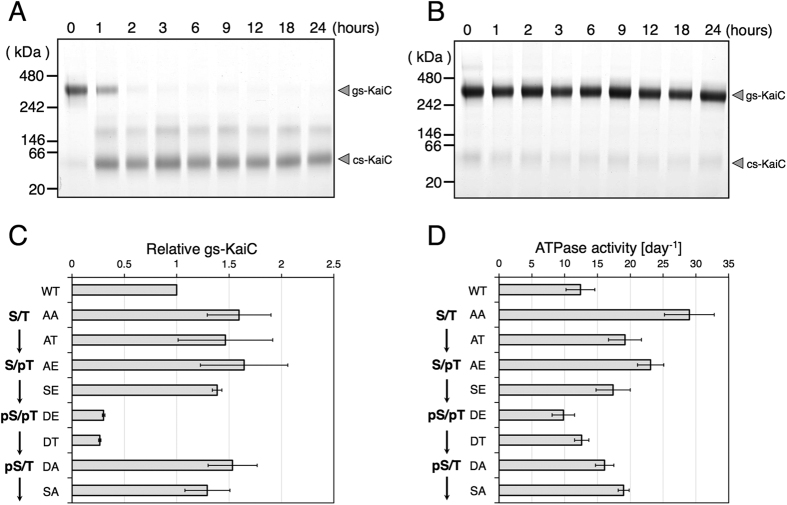
Conversion of KaiC conformational states. KaiC-DE (S431D, T432E) (**A**) and KaiC-AA (S431A, T432A) (**B**) were incubated in the presence of ATP, and the reaction mixtures were subjected to BN-PAGE. (**C**) The eight phosphorylation state-mimicking KaiC variants were incubated in the presence of ATP at 30 °C for 24 h, and were then subjected to BN-PAGE analyses (Fig. S5). Quantities of gs-KaiC in the series of phosphorylation state-mimicking variants were plotted as relative band densities in BN-PAGE analyses of wild-type (WT) KaiC, and are listed in order of the sequences of their phosphorylation mimicking forms in the phosphorylation cycle of KaiC, as indicated in the leftmost labels. The data represent the means ± SD from three independent experiments. (**D**) ATPase activities (moles_ADP_/KaiC/day) of a series of phosphorylation state-mimicking KaiC variants were measured using HPLC. The data represent the means ± SD from three independent experiments.

During the KaiC phosphorylation cycle, Ser431 and Thr432 residues in the CII-domain are autophosphorylated and autodephosphorylated in a programmed sequence as follows: S/T → S/pT → pS/pT → pS/T → S/T, where ‘S’ represents Ser431, ‘pS’ represents phosphorylated-Ser431, ‘T’ represents Thr432 and ‘pT’ represents phosphorylated-Thr432^15^. Because the appearance of the cs-KaiC band under clock-reconstituted conditions was strongly correlated with phosphorylation levels of KaiC in BN-PAGE analysis (Fig. 1), we investigated the effects of KaiC phosphorylation on interconversion between the two conformational states of KaiC using recombinant KaiC variants that mimic each phosphorylation state. In these experiments, we generated a phosphorylated variant (KaiC-DE) in which Ser431 and Thr432 residues were replaced with aspartate and glutamate (S431D and T432E). In subsequent BN-PAGE analyses, almost all KaiC-DE was detected as the gs-KaiC band at 0 h and was rapidly converted to the cs-KaiC band within 3 h at 30 °C (Fig. 3A). Moreover, similar to wild-type KaiC, KaiC-DE was eluted as a single peak in gel filtration chromatography analyses throughout the 24 h cycle, confirming that the hexameric structure of KaiC-DE is as stable as that of the wild type in the absence of CBB (Fig. S4A). In further experiments, we generated a dephosphorylated state-mimicking variant (KaiC-AA) in which Ser431 and Thr432 were replaced with alanine (S431A and T432A). In BN-PAGE experiments, the KaiC-AA variant migrated at the position of the gs-KaiC hexamer throughout the 24-h incubation (Fig. 3B) and showed a single peak of the hexamer in gel filtration chromatography, as observed with wild-type KaiC (Fig. S4B). These observations indicate that KaiC-AA stably maintains hexameric structure in the presence of CBB and that conformations of the KaiC-DE and KaiC-AA variants are found in the cs-KaiC and gs-KaiC states at 30 °C, respectively.

In analyses of a series of KaiC variants that mimicked individual phases of circadian KaiC-phosphorylation (Fig. 3C), we constructed variants by introducing S431A, S431D, T432A, T432E, S431A/T432E and S431D/T432A substitutions to wild-type KaiC and named the variants KaiC-AT, KaiC-DT, KaiC-SA, KaiC-SE, KaiC-AE and KaiC-DA, respectively. Relative quantities of gs-KaiC were estimated in each variant using densitometry of the bands separated on BN-PAGE gels (Fig. 3C, S5). Only KaiC-DE and KaiC-DT were found in the cs-KaiC state and the other variants were in the gs-KaiC state. Hence, phosphorylation of Ser431 is required for the conversion from gs-KaiC to cs-KaiC.

### ATP hydrolysis of the CI-domain induces cs-KaiC

To determine whether ATPase activities are related to conformational changes of KaiC, we initially correlated ATPase activities with phosphorylation states of KaiC by measuring ATPase activities of phosphorylation state-mimicking KaiC variants (Fig. 3D). In these experiments, KaiC-AA and KaiC-DE showed the highest and lowest activities, respectively. Moreover, among a subset of variants (AA, AT, AE, SE and DE) that mimic auto-phosphorylating KaiCs, ATPase activities in descending order were consistent with corresponding phosphorylation states in the auto-phosphorylating reaction sequence (S/T → S/pT → pS/pT), except for KaiC-AT (Fig. 3D). In addition, experiments with the subset of variants (DE, DT, DA and SA) that mimic auto-dephosphorylating KaiCs, ATPase activities in ascending order were consistent with the order of phosphorylation states in the auto-dephosphorylating reaction sequence (pS/pT → pS/T → S/T; Fig. 3D). Taken together, these data indicate that ATPase activities of phosphorylation state-mimicking variants are strongly correlated with the phosphorylation and dephosphorylation states of KaiC.

**Figure 4:**
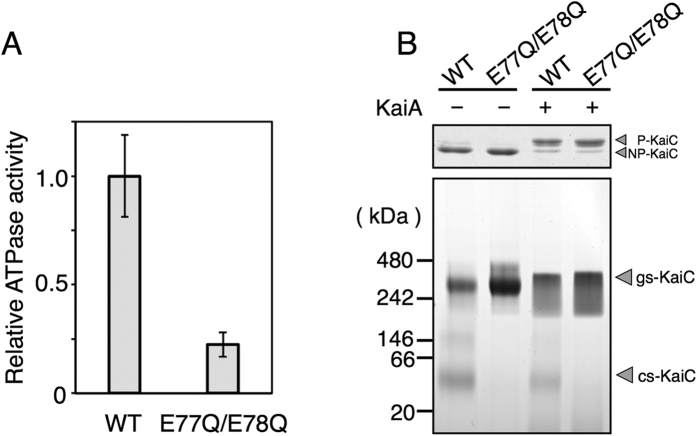
(**A**) ATPase activity of E77Q/E78Q-KaiC relative to that of wild-type (WT) KaiC. The data represent the means ± SD from three independent experiments. (**B**) E77Q/E78Q-KaiC was incubated in the presence or absence of KaiA at 30 °C for 24 h, and was then subjected to SDS-PAGE (upper panel) and BN-PAGE (lower panel) analyses.

Because ATPase activities and phosphorylation states were correlated with each other, we examined which of these contributes to the conformational change of KaiC by analysing the KaiC variant E77Q/E78Q-KaiC, in which conserved catalytic glutamate residues in the CI-domain were substituted for glutamine residues. ATPase activities of E77Q/E78Q-KaiC were only 22% of wild-type KaiC activity (Fig. 4A), confirming that Glu77 and Glu78 are crucial for ATPase activities of the CI-domain, as previously reported^9^,^28^. In contrast, phosphorylation of this variant and wild type was stimulated by KaiA (Fig. 4B, upper panel), showing that the phosphorylation activities are fully retained in the KaiC variant even though the ATPase activity derived from the CI-domain was impaired, as previously reported^20^. In BN-PAGE experiments, E77Q/E78Q-KaiC remained in the gs-KaiC state and no cs-KaiC state was observed even after 24-h incubation at 30 °C (Fig. 4B, lower panel). Because Ser431 would be phosphorylated in the presence of KaiA under these conditions, these observations indicate that ATPase activity from the CI-domain rather than KaiC phosphorylation is essential for the conformational change from gs-KaiC to cs-KaiC.

### KaiA suppresses the state-conversion of KaiC

**Figure 5:**
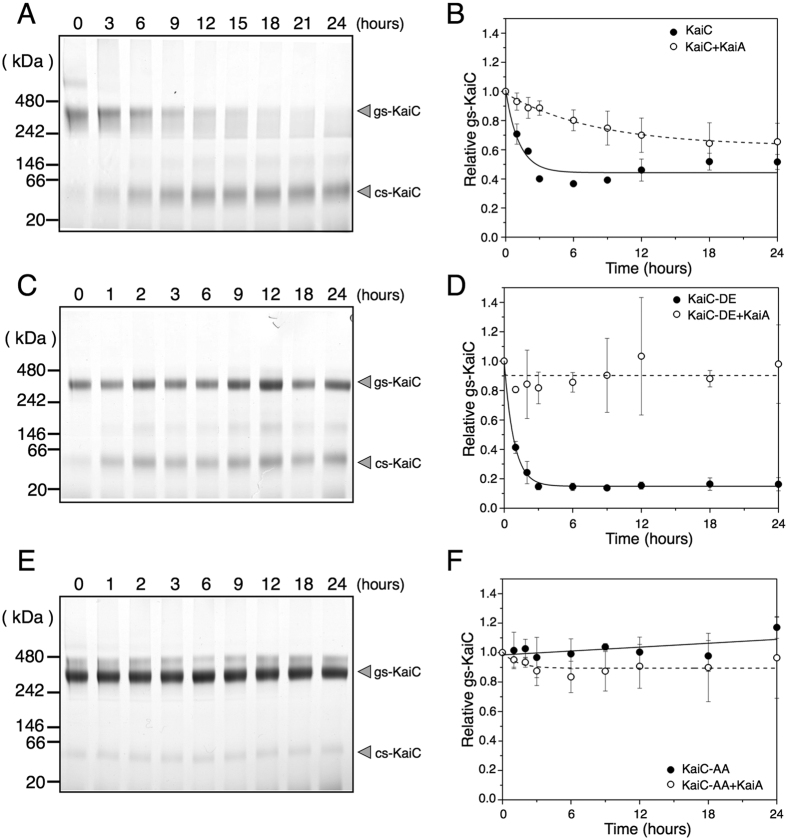
Repression of conversion of the KaiC conformation by KaiA. KaiC-WT (**A**), KaiC-DE (**C**) and KaiC-AA (**E**) were incubated in the presence of KaiA at 30 °C and were then subjected to BN-PAGE. (**B,D,F**) Band densities of gs-KaiC relative to that at 0 h were plotted against time. Open circles (○) show time courses of gs-KaiC in the presence of KaiA (Fig. 5A,C,E), and black circles (●) indicate incubation without KaiA (Figs 2A and 3A,B). Kinetics for all reactions was fitted with mono-exponential decay functions including an offset (y = y_0_ + Ae^−x/t^) using the Levenberg–Marquardt algorithm. Fitted curves are shown as solid and broken lines for kinetics without and with KaiA, respectively. The data represent the means ± SD from more than three independent experiments.

To investigate the effects of KaiA on conformational changes of KaiC, KaiC was incubated with KaiA at 30 °C and its conformation states were analysed using BN-PAGE. During the 24-h incubation, the gs-KaiC band of the wild-type KaiC gradually disappeared, and the cs-KaiC band concomitantly appeared (Fig. 5A), showing that the conversion from gs-KaiC to cs-KaiC proceeded as in the absence of KaiA. However, the conversion rate from gs-KaiC to cs-KaiC was approximately 7-fold slower in the presence of KaiA than in the absence of KaiA (Fig. 5B), suggesting that KaiA sustains the gs-KaiC state and represses the conformational change of KaiC.

To determine the effects of KaiC phosphorylation on KaiA activities, the phosphorylated state-mimicking variant KaiC-DE, which is converted into cs-KaiC in the absence of KaiA at 30 °C (Fig. 3A), was incubated with KaiA at 30 °C prior to BN-PAGE analyses (Fig. 5C). In these experiments, KaiA inhibited the conformational change of KaiC-DE, as indicated by total arrest of the rapid (within 6 h) and complete conversion of KaiC-DE from the gs-KaiC state into the cs-KaiC state (Fig. 5D). The dephosphorylated state-mimicking variant KaiC-AA, which was fixed as gs-KaiC in the absence of KaiA (Fig. 3B), remained in the gs-KaiC state regardless of the presence or absence of KaiA (Fig. 5E,F). These data suggest that KaiA suppresses the conversion from gs-KaiC to cs-KaiC.

### KaiB binds to cs-KaiC

**Figure 6:**
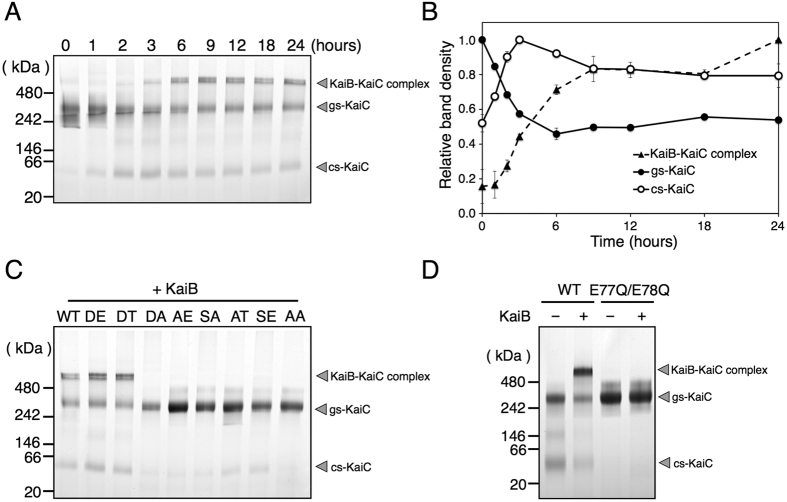
Specific binding of KaiB to cs-KaiC. (**A**) KaiC was incubated with KaiB at 30 °C, and reaction mixtures were subjected to BN-PAGE. The upper band represents a KaiB-KaiC complex and the intermediate and lower bands correspond with gs-KaiC and cs-KaiC. (**B**) Densitometric quantitation of (**A**); The plots show gs-KaiC (●), cs-KaiC (○) and the complex (▲) at each time point. Maximum densities of the each band through the incubation (gs-KaiC at time 0 h, cs-KaiC at 3 h and the complex at 24 h) were assigned a value of 1. The data represent the means ± SD from three independent experiments. (**C**) Phosphorylation state-mimicking KaiC variants were incubated with KaiB at 30 °C for 12 h, and reaction mixtures were subjected to BN-PAGE analyses. (**D**) The ATPase variant of KaiC was incubated with or without KaiB at 30 °C for 12 h, and reaction mixtures were subjected to BN-PAGE analyses.

To determine whether conformational changes of KaiC affect binding of KaiB to KaiC, KaiC was incubated with KaiB at 30 °C and was then subjected to BN-PAGE (Fig. 6A). During the first 3 h, cs-KaiC levels increased with decreases in gs-KaiC levels (Fig. 6B). In addition to gs-KaiC and cs-KaiC bands, an additional band was observed with a higher molecular weight than gs-KaiC (Fig. 6A). This band was subsequently identified as a KaiB–KaiC complex in immunoblotting experiments (Fig. S2) and appeared at the beginning of the incubation and gradually increased until 9 h. Moreover, gs-KaiC levels remained constant after 6 h, whereas cs-KaiC levels gradually decreased with increases in the KaiB–KaiC complex (Fig. 6B), suggesting that gs-KaiC is initially converted into cs-KaiC and then KaiB binds to cs-KaiC giving rise to the KaiB–KaiC complex. Hence, KaiB specifically binds cs-KaiC but not gs-KaiC, and the conformational change from gs-KaiC to cs-KaiC may be a trigger for the binding of KaiB.

To confirm this hypothesis, a series of phosphorylation-state mimicking variants of KaiC were incubated with KaiB at 30 °C, and KaiB–KaiC complex formation was analysed using BN-PAGE (Fig. 6C). In these experiments, the KaiB–KaiC complex was detected only in the variants KaiC-DE and KaiC-DT (Fig. 6C), as wild-type KaiC. These two variants are coincident with those existed in the cs-KaiC state during the single incubation shown in Fig. 3C. In contrast, the KaiB–KaiC complex was not detected in the other variants (Fig. 6C) or the ATPase-deficient variant E77Q/E78Q-KaiC (Fig. 6D) as previously shown^20^. They all were fixed in the gs-state (Figs 3C and 4B). These results clearly demonstrated that KaiB binds specifically to KaiC in the cs-KaiC state and forms the KaiB–KaiC complex.

## Discussion

In the present study, we demonstrated gs-KaiC and cs-KaiC conformational states of KaiC hexamers using BN-PAGE analyses. BN-PAGE is widely used to identify protein complexes and estimate oligomeric states of protein complexes and apparent molecular masses^25^,^26^. In BN-PAGE analyses, electrophoretic mobility was conferred to each protein at a constant charge/mass ratio by non-specific hydrophobic binding of the amphipathic anion CBB dye. Most separated proteins on BN-PAGE gels retained their higher-order structures, although several examples of denaturation of protein complexes have been documented even under the less destructive conditions of BN-PAGE^29^. In the present study, we found that the cs-KaiC hexamer readily dissociates into monomers by binding CBB, whereas gs-KaiC was tightly assembled and was tolerant of CBB binding (Fig. 2). These differing states of KaiC have not been identified in previous spectroscopic studies, including those reporting NMR and SAXS, and X-ray crystallography data. Hence, BN-PAGE analysis is a powerful and effective technique for detecting conformational differences in KaiC hexamers and Kai protein complexes.

Binding of CBB caused dissociation of cs-KaiC into monomers. Although we investigated the effects of both CBB and high concentrations of a chaotrophic ion (Cl^−^, up to 1 M) and four detergents (Fig. S3), only CBB caused hexamer dissociation (Fig. S3). CBB is commonly used to stain proteins in gel electrophoresis, and for determinations of protein concentrations. However, CBB may cause dissociation of unstable protein complexes by introducing negative charges to subunit interfaces of complexes, causing dissociation of complexes via electrostatic repulsions between subunits^29^. Assuming that the conformation of cs-KaiC is more flexible and labile than that of gs-KaiC, CBB may intercalate into protomer interfaces of cs-KaiC hexamers, but not to those of gs-KaiC, in which protomers are tightly assembled. Because CBB has been shown to interact strongly with arginine residues^30^, five arginine residues in protomer interfaces of the CI-domain, such as Arg40, Arg161, Arg215, Arg216 and Arg226 may be responsible for CBB-dependent dissociation of cs-KaiC hexamers. In the present BN-PAGE experiments, the gs-KaiC band was diffuse and broad, and only its lower side was converted to the cs-KaiC band (Fig. 2A). In addition, a few faint bands and two major bands corresponding to gs-KaiC and cs-KaiC appeared in native-PAGE analysis after addition of CBB to KaiC samples (Fig. 2C). These results suggest the presence of several sub-structures of gs-KaiC that can bind varying numbers of CBB molecules, and some of these may be converted into cs-KaiC.

Interconversions between gs-KaiC and cs-KaiC were strongly correlated with both phosphorylation states and ATPase activities of KaiC (Fig. 3). Whereas cs-KaiC was converted from the Ser431-phosphorylated gs-KaiC (Fig. 3C), observations of E77Q/E78Q-KaiC indicated that rather than the phosphorylation of KaiC, ATP hydrolysis in the CI-domain is a direct trigger for the conversion from gs-KaiC to cs-KaiC (Fig. 4). Hence, conformational differences between cs-KaiC and gs-KaiC may predominantly reflect the CI-domain of KaiC.

The present data show that KaiB only binds cs-KaiC (Fig. 6). The conversion from gs-KaiC into cs-KaiC requires phosphorylation of Ser431 in the CII-domain (Fig. 3C) and ATP-hydrolysis in the CI-domain (Fig. 4). Therefore, the conversion into cs-KaiC would be a result of structural interaction between CI and CII rings of a KaiC hexamer. Previous reports showed that stacking interaction between CI and CII rings is necessary for KaiB-KaiC complex formation^31^, and KaiB binds to CI-domain^31^,^32^. However, further studies are needed to assess the structure corresponding to the gs-KaiC and cs-KaiC in the course of structural changes of CI and CII rings.

In the present BN-PAGE experiments, KaiC-DT and KaiC-DE bound to KaiB among the phosphorylation state-mimicking variants (Fig. 6C). KaiB did not form stable complexes with KaiC-DA, which is consistent with a previous report^33^. In contrast, another report has shown that KaiB can bind to KaiC-EA, which is considered to mimic the same phosphorylation stage of KaiC as KaiC-DA^34^. These contradictory results may arise from the side chain structure at the 431 position of KaiC. The side chain of glutamate of KaiC-EA is longer than that of aspartate of KaiC-DA by one alkyl carbon, which could cause some steric effect on the surrounding structure.

Previously, a threshold of KaiC hexamer phosphorylation was proposed to control the change to the hexameric configuration and the affinity for KaiB^15^,^20^. In our data, the phosphorylation state of KaiC strongly correlated with its ATPase activity (Fig. 3D), and ATP hydrolysis in the CI-domain was required for conversion to the cs-KaiC configuration (Fig. 4), suggesting that KaiB-binding is directly triggered by ATP hydrolysis inducing conformational change of the gs-KaiC to cs-KaiC, rather than phosphorylation of Ser431. Moreover, in a recent study of KaiB–KaiC complex formation, the two homologous domains of KaiC, CI and CII, exhibited functional coupling, and phosphorylation in the CII-domain induced the ATPase reaction cycle in the CI-domain, which is required for binding of KaiB and formation of the KaiB–KaiC complex^20^. Within this framework, the phosphorylation of KaiC should increase ATPase activity of the CI-domain, although our experiments with phosphorylation state-mimicking KaiC variants clearly demonstrated that phosphorylation of KaiC decreases its ATPase activity (Fig. 3). This apparent inconsistency may be resolved by assuming that formation of the KaiB–KaiC complex requires binding of KaiC to the product of ATP hydrolysis rather than ATPase activity *per se*, which has been proposed previously^22^. Accordingly, KaiB may not bind to dephosphorylated gs-KaiC because of the rapid dissociation of the ATP hydrolysis product from the CI-domain relative to the conformational change to cs-KaiC.

**Figure 7:**
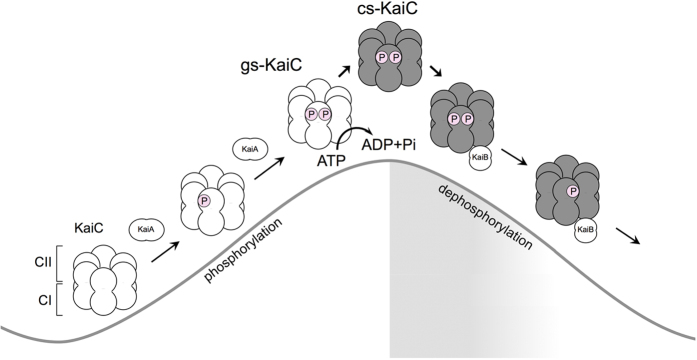
A schematic model of circadian oscillation including the conformational change of KaiC. Two conformational states of KaiC hexamers are interconverted periodically during circadian oscillations. KaiA interacts with the gs-KaiC hexamer, which stimulates phosphorylation of the CII-domain and suppresses the conversion to cs-KaiC, thus increasing phosphorylated gs-KaiC levels. After accumulation of phosphorylated Ser431 to threshold levels in each gs-KaiC hexamer, ATP hydrolysis in the CI-domain induces the conversion from gs-KaiC to cs-KaiC (gray). KaiB binds to the cs-KaiC hexamer and forms a KaiB-KaiC complex. Because of the inhibitory effects of KaiB on the KaiA-interaction, KaiC is gradually auto-dephosphorylated in the KaiB–KaiC complex.

Taken together, the present conformational change of KaiC likely contributes to circadian rhythms and promotes formation of the KaiB–KaiC complex as follows (Fig. 7): KaiA interacts with gs-KaiC and stimulates auto-phosphorylation of gs-KaiC. Subsequent phosphorylation of Ser431 in the CII-domain delays dissociation of the ATP hydrolysis product in the CI-domain. KaiC is then arrested in the ATP hydrolysis product-binding state and is converted into the cs-KaiC conformation to promote KaiB-binding. Subsequently, auto-dephosphorylation proceeds following inhibition of the KaiA interaction in the KaiB–KaiC complex, and dephosphorylation of Ser431 induces dissociation of the ATP hydrolysis product from the CI-domain. Finally, KaiC returns to the gs-KaiC conformation and KaiB dissociates from gs-KaiC to promote the KaiA-interaction again. The present conformational change of KaiC hexamers likely plays an important role as a trigger for assembly of the hetero-complex of Kai proteins in the circadian oscillator system.

## Materials and Methods

### Bacterial strains and plasmid construction for Kai proteins

*Escherichia coli* DH5α cells were used as hosts for plasmid construction and expression of Strep-tagged proteins. The genes *kaiA*, *kaiB* and *kaiC* in *S. elongatus* PCC 7942 were cloned into the *Bsa*I site of a pASK-IBA-5plus (IBA). The expression plasmid for KaiC was mutated to obtain a series of KaiC variant proteins containing the following mutations: Ser431 to aspartic acid (S431D); Thr432 to glutamic acid (T432E); Ser431 to alanine (S431A); Thr432 to alanine (T432A), Glu77 to glutamine (E77Q), and Glu78 to glutamine (E78Q) according to the Quick-Change mutagenesis protocol (Stratagene). All oligo-nucleotide primers are listed in Supplementary Table S1.

### Purification of recombinant Kai proteins

Following transformation of *E. coli* DH5α cells with expression plasmids, cells were cultured in LB medium containing 100 μg/ml ampicillin with vigorous agitation at 37 °C. At an O.D._600_ of approximately 0.5, cells were in the mid-exponential growth phase and overexpression of recombinant proteins was induced by the addition of 0.2 μg/ml anhydrotetracycline. Cells were then cultured with vigorous agitation at 37 °C and were collected at 7 h after induction. Purification was performed at 4 °C and cells expressing KaiA or KaiB were resuspended in buffer A containing 20 mM Tris–HCl (pH 8.0) and 150 mM NaCl, and were then homogenised by sonication (SONICS, VCX-130). Homogenates were centrifuged at 18,000 *g*, and supernatants were applied to a Strep-tactin Sepharose column (IBA). After adsorption of Kai proteins, columns were washed with 20-column volumes of buffer A, and were eluted with five-column volumes of buffer A containing 2.5 mM D-desthiobiotin. KaiA was further purified using ion exchange chromatography on a RESOURCE Q column (GE Healthcare) with a gradient of NaCl. To remove desthiobiotin and NaCl from final preparations, purified KaiA and KaiB were further applied to a HiPrep 16/60 Sephacryl S-200 HR column (GE Healthcare) equilibrated with buffer A. KaiC and its variants were then purified as described for KaiB, except using buffer C containing 20 mM Tris–HCl (pH 8.0), 150 mM NaCl, 1 mM ATP and 5 mM MgCl_2_ instead of buffer A throughout purification steps. During gel filtration steps, purified KaiC samples were applied to a HiPrep 16/60 Sephacryl S-300 HR column (GE Healthcare). Protein concentrations were determined according to the Bradford method using protein assay kits (Wako) with BSA (Bio-Rad) as standard.

### *In vitro* reconstitution of KaiC phosphorylation cycle

The KaiC phosphorylation cycle was reconstituted *in vitro* as previously described^4^. Briefly, KaiC was incubated with KaiA and KaiB in buffer C at 30 °C. Final concentrations of KaiA, KaiB and KaiC were 1.2, 3.5 and 3.5 μM, respectively. Prior to BN-PAGE analyse, concentrations of each Kai protein were increased twice to enhance protein signals. Phosphorylated forms of KaiC were separated using SDS–PAGE as described previously^4^, and SDS-PAGE gels were stained with Quick-CBB-plus (Wako). Relative quantities of phosphorylated KaiC in each sample were determined using densitometric analyses with Image J software^35^.

### BN-PAGE and CBB-free native PAGE

BN-PAGE and CBB-free native PAGE analyses were performed using a NativePAGE^TM^ Novex Bis-Tris Gel System (Invitrogen) according to the manufacturer’s protocols with some modifications. In brief, proteins were incubated in buffer C at 30 °C, and final concentrations of KaiA, KaiB and KaiC were 2.4, 7.0 and 7.0 μM, respectively. Aliquots of incubated proteins were suspended in sample buffer immediately prior to electrophoresis and incubated reaction mixtures were not frozen before electrophoresis. To prepare samples for BN-PAGE, 12 μl reaction mixtures were mixed with 8 μl of sample buffer containing 1× Native PAGE sample buffer (Invitrogen), 0.25% CBB-G250, 1 mM ATP and 5 mM MgCl_2_ (final concentrations). Samples were then loaded onto NativePAGE^TM^ 4–16% Bis-Tris Protein Gels (Invitrogen) and were electrophoresed. For both of the anode- and cathode-sides buffers, Native PAGE Running buffer (Invitrogen) was modified to contain 5 mM MgCl_2_ and 0.25 mM EDTA. In addition, 0.02% CBB, 1 mM ATP were added to the cathode-side buffer. The NativeMark protein standard (Invitrogen) was used as a molecular weight marker. For CBB-free native PAGE analyses, aliquots of incubated reaction mixtures in the sample buffer (1× Native PAGE sample buffer; Invitrogen) containing 1 mM ATP and 5 mM MgCl_2_ were electrophoresed in a buffer containing 192 mM Glycine, 25 mM Tris and 1 mM ATP.

### Gel filtration chromatography

Kai proteins were size-fractionated at 30 °C using a HPLC system (LC-10AVP, Shimadzu) equipped with a COSMOSIL packed column 5Diol-300-II (Nacalai Tesque) equilibrated with buffer C. Proteins were eluted at a flow rate of 0.4 ml/min and were monitored spectrophotometrically at 280 nm using a UV detector (APD-10AVP, Shimadzu). Molecular mass standards for gel filtration analyses included apoferritin (443 kDa), β-amylase (200 kDa), alcohol dehydrogenase (150 kDa), serum albumin (66 kDa) and carbonic anhydrase (29 kDa; Sigma-Aldrich).

### ATPase activity measurement

ATPase activities of KaiC were measured using a HPLC system (LC-10AVP, Shimadzu) equipped with a Shim-Pack VP-ODS column (Shimadzu). ATP and ADP in reaction mixtures were separated using column at 30 °C with a flow rate of 0.4 ml/min and were detected at 260 nm. The mobile phase comprised 100 mM phosphoric acid, 150 mM triethylamine and 1% acetonitrile. ATPase activities were evaluated as a function of ADP production.

## Additional Information

**How to cite this article**: Oyama, K. *et al*. Conversion between two conformational states of KaiC is induced by ATP hydrolysis as a trigger for cyanobacterial circadian oscillation. *Sci. Rep.* **6**, 32443; doi: 10.1038/srep32443 (2016).

## Supplementary Material

Supplementary Information
