# Supplementary material for: Conversion between two conformational states of KaiC is induced by ATP hydrolysis as a trigger for cyanobacterial circadian oscillation
Source: Sci Rep. 2016 Sep 1;6:32443. doi: 10.1038/srep32443 (PMC5007536; doi:10.1038/srep32443)
Supplement: Supplementary Information [file srep32443-s1.pdf]

Supplementary Information

Conversion between two conformational states of KaiC is induced by ATP hydrolysis as a trigger for cyanobacterial circadian oscillation

Katsuaki Oyama, Chihiro Azai, Kaori Nakamura, Syun Tanaka and Kazuki Terauchi*

*To whom correspondence should be addressed. E-mail: terauchi@fc.ritsumei.ac.jp.

Supplementary Materials and Methods

Western blotting

Purified strep-KaiA or strep-KaiB were used to immunize rabbits, and antisera against KaiA or KaiB were isolated. Antibody specificities were confirmed by Western blotting using the purified proteins as a control. Antiserum specifically detected KaiA or KaiB proteins with molecular masses of approximately 34 and 13 kDa, respectively.

To detect KaiA, BN-PAGE gels were equilibrated with buffer containing 50 mM Tris–HCl (pH 6.8), 6 M urea, 30% glycerol, 2% SDS and 16 mM DTT. Cut-off pieces of the gel were subjected to SDS-PAGE, and proteins were transferred to PVDF membranes, followed by incubation with anti-KaiA antisera at a 1:100,000 dilution. To detect KaiB, BN-PAGE gels were soaked in 100% methanol, and proteins of BN-PAGE gels were transferred to PVDF membranes followed by incubation with anti-KaiB antisera at a 1:1,000 dilution. Membranes were incubated with horseradish peroxidase-conjugated anti-rabbit IgG (Bio-Rad) at a 1:5,000 dilution, and the proteins were then detected using chemiluminescence assay reagents (Chemi-Lumi One Super, Nacalai tesque) and an ImageQuant LAS 4000 image analyser (Fujifilm) for KaiA, and a Molecular Imager ChemiDoc XRS+ (BioRad) for KaiB.

Supplementary Table S1. primers for mutagenesis of plasmids

Mutant	Primers
KaiC-AA	5'-GACTCCCATATCGCAATTACGGATACG-3'<br>5'-CGTATCCGTAATTGCTGCGATATGGGAGTC-3'
KaiC-DE	5'-ATTACTGACTCCCATATCGATGAAATTACG-3'<br>5'-CGTAATTTCATCGATATGGGAGTCAGTAAT-3'
KaiC-AE	5'-TCCCATATCGCAGAAATTACGGAT-3'<br>5'-ATCCGTAATTTCTGCGATATGGGA-3'
KaiC-SE	5'-TCCCATATCTCAGAAATTACGGAT-3'<br>5'-ATCCGTAATTTCTGAGATATGGGA-3'
KaiC-DT	5'-TCCCATATCGATACAATTACGGAT-3'<br>5'-ATCCGTAATTGTATCGATATGGGA-3'
KaiC-DA	5'-TCCCATATCGATGCAATTACGGAT-3'<br>5'-ATCCGTAATTGCATCGATATGGGA-3'
KaiC-SA	5'-CTCCCATATCTCAGCAATTACGGATACG-3'<br>5'-CGTATCCGTAATTGCTGAGATATGGGAG-3'
KaiC-AT	5'-CTCCCATATCGCAACAATTACGGATACG-3'<br>5'-CGTATCCGTAATTGTTGCGATATGGGAG-3'
E77Q/E78Q	5'-CAAACCCCGCAAGATATCATTA-3'<br>5'-TTGGAAAGTAACGAAAACCCCAG -3'

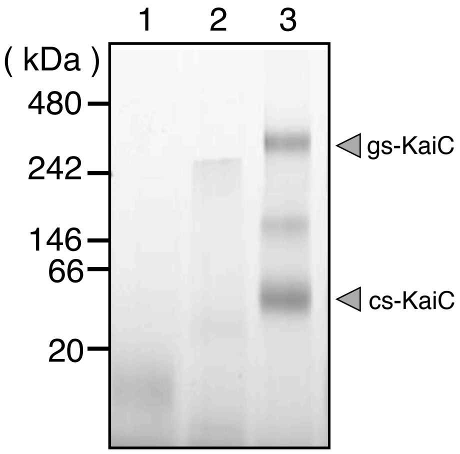

Fig. S1. BN-PAGE of Kai proteins

Oligomeric states of purified KaiA, KaiB and KaiC proteins were analysed using BN-PAGE after incubation of purified proteins for 9 h at 30°C. Neither KaiA nor KaiB were detected clearly, with very faint bands (lane 1, 2) that had unexpected electrophoretic mobility. The purified KaiC protein was detected as two major bands with apparent molecular weights of upper and lower bands that represented hexameric and monomeric forms of KaiC, respectively (lane 3). Upper and lower bands were designated gs-KaiC and cs-KaiC, respectively.

Previous crystallographic studies indicate that KaiA (monomer = 32.6 kDa) assembles as a dimer (ref. 1), KaiB (monomer = 11.5 kDa) assembles as a dimer or a tetramer (ref. 2, 3) and KaiC (monomer = 58 kDa) assembles as a hexamer (ref. 4).

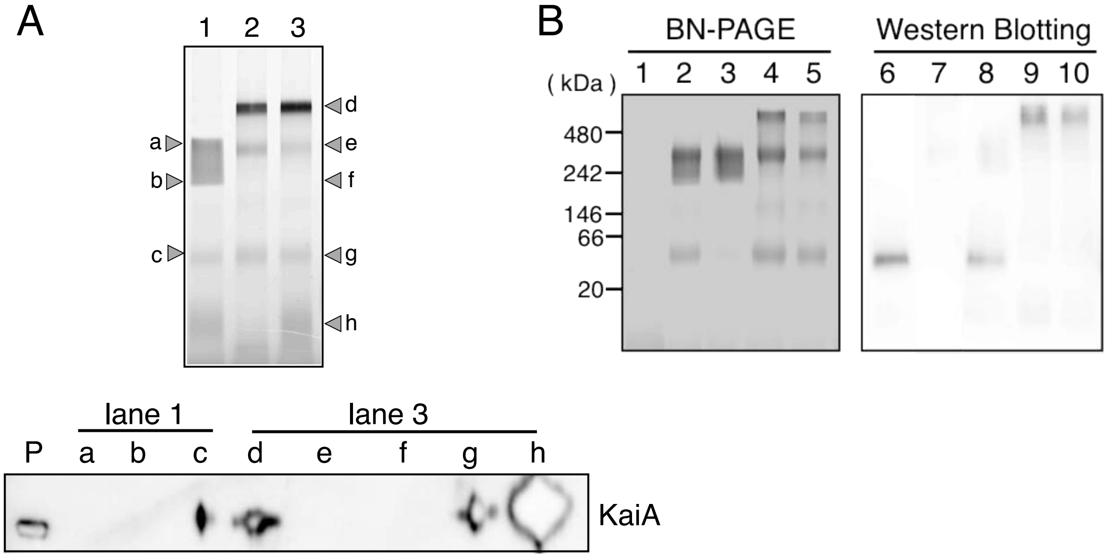

Fig. S2. Detection of KaiA and KaiB proteins

(A) BN-PAGE analyses of reaction mixtures of KaiA+KaiC (lane 1), KaiB+KaiC (lane 2) and KaiA+KaiB+KaiC (lane 3; upper panel). Reaction mixtures were incubated in the presence of ATP at 30°C for 9 h, and were subjected to BN-PAGE. Proteins in respective bands (a-h) were extracted from BN-PAGE gels and KaiA was detected using immunoblot analyses. Cut-out pieces were examined using 2D electrophoresis and Western blotting with anti-KaiA antibody (lower panel). Purified KaiA protein was used as a control (P).

(B) BN-PAGE of KaiB (lane 1), KaiC (lane 2) and KaiC following incubation with KaiB at 30°C for 0 (lane 3), 12 (lane 4) and 24 h (lane 5). Western blotting (right panel) was performed after BN-PAGE (left panel); KaiB (lane 6), KaiC (lane 7), KaiB+KaiC at 0 h (lane 8), KaiB+KaiC at 12 h (lane 9) and KaiB+KaiC at 24 h (lane 10).

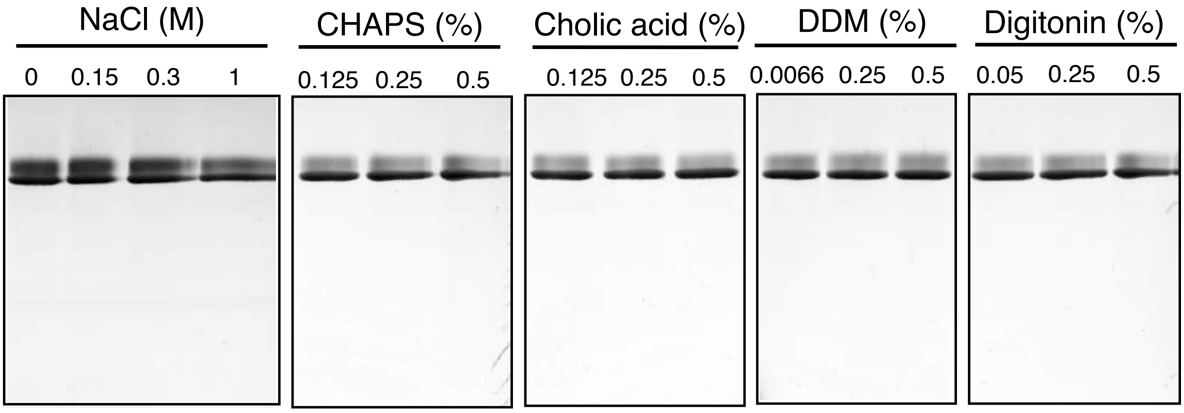

Fig. S3. Effects of detergents and NaCl on KaiC

KaiC was incubated in the presence of ATP at 30°C for 12 h, and was then subjected to native-PAGE in the presence of NaCl at 150, 300 or 1000-mM, CHAPS at 0.125%, 0.25% or 0.5%, cholic acid at 0.125%, 0.25% or 0.5 %, dodecyl maltoside (DDM) at 0.0066%, 0.25% or 0.5% or Digitonin at 0.05%, 0.25% or 0.5%.

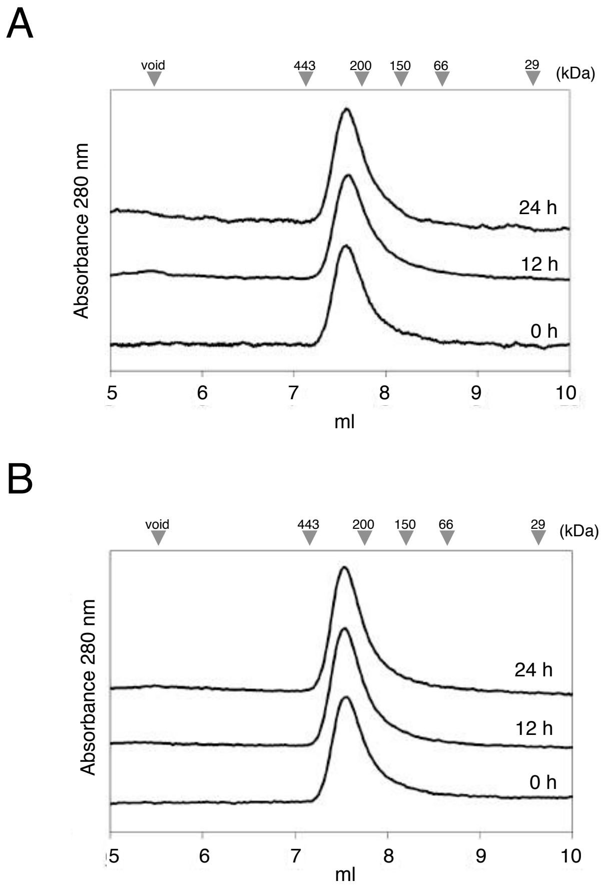

Fig. S4. Elution profiles from gel filtration chromatography of KaiC-DE (A) and KaiC-AA (B) at 0, 12 and 24 h

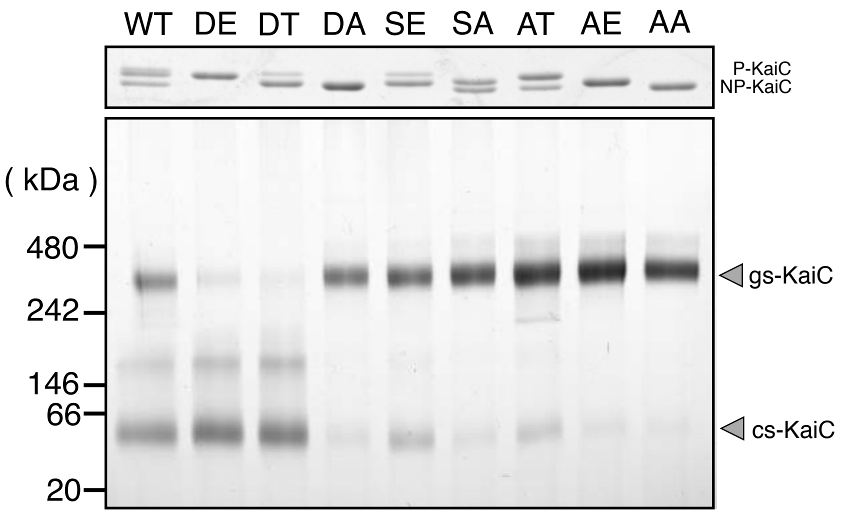

Fig. S5. BN-PAGE of phosphorylation state-mimicking KaiC variants

Eight phosphorylation state-mimicking KaiC variants were incubated in the presence of ATP at 30°C for 24 h, and were then subjected to SDS-PAGE (upper panel) and BN-PAGE (lower panel) analyses.

Supplementary References

1. Ye, S., Vakonakis, I., Ioerger, T. R., LiWang, A. C. & Sacchettini, J. C. Crystal structure of circadian clock protein KaiA from *Synechococcus elongatus*. *J Biol Chem* **279**, 20511-20518 (2004).
2. Iwase, R. *et al.* Functionally important substructures of circadian clock protein KaiB in a unique tetramer complex. *J Biol Chem* **280**, 43141-43149 (2005).
3. Villarreal, S. A. *et al.* CryoEM and molecular dynamics of the circadian KaiB-KaiC complex indicates that KaiB monomers interact with KaiC and block ATP binding clefts. *J Mol Biol* **425** (2013).
4. Pattanayek, R. *et al.* Visualizing a circadian clock protein: crystal structure of KaiC and functional insights. *Mol Cell* **15**, 375-388 (2004).
